# Phylogenetic analysis of the SINA/SIAH ubiquitin E3 ligase family in *Metazoa*

**DOI:** 10.1186/s12862-017-1024-x

**Published:** 2017-08-07

**Authors:** Ian J. Pepper, Robert E. Van Sciver, Amy H. Tang

**Affiliations:** Department of Microbiology and Molecular Cell Biology, Eastern Virginia Medical School, Leroy T. Canoles Jr. Cancer Research Center, Harry T. Lester Hall, Room 454-457, 651 Colley Avenue, Norfolk, VA 23501 USA

**Keywords:** RAS signal transduction, SINA/SIAH E3 ligases, Ubiquitin-mediated proteolysis, Phylogenetic analysis, Invariant and divergent amino acid residues, And conserved functional domains in SINA, SIAH1, SIAH2 and SIAH3

## Abstract

**Background:**

The RAS signaling pathway is a pivotal developmental pathway that controls many fundamental biological processes including cell proliferation, differentiation, movement and apoptosis. *Drosophila* Seven-IN-Absentia (SINA) is a ubiquitin E3 ligase that is the most downstream signaling “gatekeeper” whose biological activity is essential for proper RAS signal transduction. Vertebrate SINA homologs (SIAHs) share a high degree of amino acid identity with that of *Drosophila* SINA. SINA/SIAH is the most conserved signaling component in the canonical EGFR/RAS/RAF/MAPK signal transduction pathway.

**Results:**

Vertebrate SIAH1, 2, and 3 are the three orthologs to invertebrate SINA protein. SINA and SIAH1 orthologs are found in all major taxa of metazoans. These proteins have four conserved functional domains, known as RING (Really Interesting New Gene), SZF (SIAH-type zinc finger), SBS (substrate binding site) and DIMER (Dimerization). In addition to the *siah1* gene, most vertebrates encode two additional *siah* genes (*siah2* and *siah3*) in their genomes. Vertebrate SIAH2 has a highly divergent and extended N-terminal sequence, while its RING, SZF, SBS and DIMER domains maintain high amino acid identity/similarity to that of SIAH1. But unlike vertebrate SIAH1 and SIAH2, SIAH3 lacks a functional RING domain, suggesting that SIAH3 may be an inactive E3 ligase. The SIAH3 subtree exhibits a high degree of amino acid divergence when compared to the SIAH1 and SIAH2 subtrees. We find that SIAH1 and SIAH2 are expressed in all human epithelial cell lines examined thus far, while SIAH3 is only expressed in a limited subset of cancer cell lines.

**Conclusion:**

Through phylogenetic analyses of metazoan SINA and SIAH E3 ligases, we identified many invariant and divergent amino acid residues, as well as the evolutionarily conserved functional motifs in this medically relevant gene family. Our phylomedicinal study of this unique metazoan SINA/SIAH protein family has provided invaluable evolution-based support towards future effort to design logical, potent, and durable anti-SIAH-based anticancer strategies against oncogenic K-RAS-driven metastatic human cancers. Thus, this method of evolutionary study should be of interest in cancer biology.

**Electronic supplementary material:**

The online version of this article (doi:10.1186/s12862-017-1024-x) contains supplementary material, which is available to authorized users.

## Background

The RAS signaling pathway in metazoans is one of the most fundamental and evolutionarily conserved signaling pathways controlling cell proliferation, motility, and stem cell renewal during normal development, tissue regeneration, and pathogenesis [1–5]. Aberrant EGFR/HER2/K-RAS pathway hyperactivation is known to promote hyperplastic growth, tumorigenesis, and metastasis [6, 7]. Therefore, inhibiting oncogenic K-RAS pathway activation is a logical strategy to stop tumor progression and prevent metastasis in human cancer [8–10]. However, the design of long-lasting anti-K-RAS therapies has remained elusive for past 40 years due to unique RAS kinetics, extensive pathway cross-talk, signal bifurcation, compensatory activation, feedback control, context-dependent adaptation and dynamic rewiring of the ERBB/K-RAS signaling network [11, 12]. Hence, alternative anti-K-RAS strategies are urgently needed to shutdown “undruggable” oncogenic K-RAS activation and eradicate oncogenic K-RAS-driven metastatic cancer in the clinic [10].

Many key RAS signaling components were identified successfully in *Drosophila* via genetic modifier screens, including Sevenless (SEV, the *Drosophila* homolog of mammalian EGFR membrane receptor), rat sarcoma viral oncogene (RAS), RAF serine/threonine kinase, Mitogen-activated Protein Kinase (MAPK), Son of Sevenless (SOS), and Seven-In-Absentia (SINA) [2, 13–19]. *Drosophila* SINA was identified as the most downstream signaling component in the *Drosophila* RAS signaling pathway, playing a critical “gatekeeper” role in R7 photoreceptor cell fate determination [2, 10, 14, 20]. Interestingly, the loss of the R7 photoreceptor mutant phenotypes observed in *sina* ^loss-of-function^ mutant flies is identical to that observed in *sev* ^loss-of-function^ mutant flies [13, 14]. SINA-mediated degradation of a neuronal repressor, Tramtrack (TTK^88^), is required to unleash active RAS signaling to initiate the neuronal cell differentiation program in Drosophila R7 precursor cells [20]. Among all the signaling components identified thus far in the RAS pathway, Drosophila SINA and human SIAH1/2 share the highest level of evolutionary conservation and amino acid identities [14, 21]. Extensive genetic epistasis analyses have demonstrated that proper SINA function is critical for RAS signal transduction, and that active EGFR, RAS, RAF, and MAPK signals cannot be transmitted properly without functional SINA. This suggests that SINA is the most downstream signaling “gatekeeper” identified thus far in the RAS signaling pathway and it is a key signaling hub critical for transmitting EGFR/RAS/RAF/MAPK activation signals in vivo [2, 17, 22].

*Drosophila* SINA and two of its human homologs, SIAH1 and SIAH2, belong to a highly evolutionarily conserved family of RING domain E3 ligases [20, 21, 23]. SINA and SIAH function as homo- and hetero-dimers [24–27]. The family of the SINA/SIAH1/SIAH2 E3 ligases has four highly conserved and distinct functional domains: (1) the Really Interesting New Gene (RING) domain is the catalytic active site for the E3 ligase activity, (2) the SIAH-type zinc finger (SZF) domain contains a dual zinc-finger motif, (3) the substrate-binding site (SBS) recognizes substrates, and (4) the dimerization (DIMER) domain allows for homo- and heterodimer formation between SINA/SIAH proteins [23–31]. The substrate-binding domain (SBD), composed of SZF, SBS and DIMER domains, is responsible for substrate recognition, targeting, interaction, and degradation [23, 25–31]. As an E3 ligase, SINA/SIAH is known to interact, modify, and target a multitude of substrates/partners/regulators to orchestrate ubiquitin-mediated proteolysis and regulate protein stability, protein complex assembly, protein subcellular localization, and other cellular functions in normal development and human diseases [10, 32, 33]. SIAH3 is a newly identified member of the vertebrate SIAH family [34, 35]. Although SIAH3 has been reported to function as a negative regulator of Parkin protein translocation within the mitochondria, SIAH3 biochemical function remains largely understudied in mammalian systems [23, 34].

Controlling oncogenic K-RAS-driven metastatic cancer remains an unmet need in medicine [8, 12]. Based on the highly conserved molecular principles and regulatory mechanisms learned from the *Drosophila* RAS signaling pathway, we have proposed and demonstrated the efficacy of a novel antitumor strategy to inhibit the “undruggable” oncogenic K-RAS signal at its most downstream signaling hub. This was achieved by inhibiting SIAH1/2 E3 ligases, using both in vitro and in vivo tumor models of human pancreatic and lung cancer [36, 37]. We and others have shown that blocking SIAH1/2 activity is a promising and logical strategy to inhibit oncogenic K-RAS/B-RAF activation and to impede oncogenic K-RAS/B-RAF-driven tumorigenesis in preclinical xenograft models [32, 36–38]. In the current study, we conducted a phylogenetic analysis of the SINA/SIAH family of E3 ligases across the entire animal kingdom to further delineate SINA/SIAH biological function by focusing on the evolutionary conservation and mutational constraints observed in this family of SINA/SIAH E3 ligases. We identified invariant and divergent amino acid residues, as well as several highly conserved functional motifs in the SINA/SIAH family. This provides an evolutionary, structural, functional, and translational basis with which to design more potent and long-lasting anti-SIAH-based anti-K-RAS strategies against oncogenic K-RAS-driven metastatic human cancers in the future.

## Results

### Phylogenetic analysis of the SINA/SIAH family of E3 ligases

**Fig. 1:**
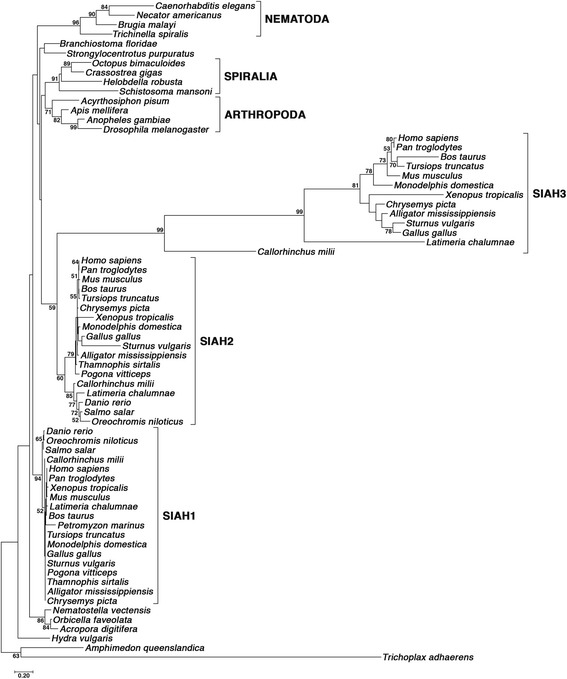
Phylogenetic tree of the evolutionarily conserved SINA/SIAH family across metazoan species. The phylogenetic tree was constructed to illustrate the evolutionary relationships of SINA/SIAH family using the representative species from all the major taxa across the entire metazoan kingdom. The LG + G4 + F model was utilized for construction of the tree. The numbers listed on each node represent the bootstrap support value associated with that node after running 100 replicates. All bootstrap values < 50 were eliminated from the tree display. The tree was manually rooted at the node containing the outgroup sequences *A. queenslandica* and *T. adhaerens*. Major clades that were recovered by the analysis are indicated by the brackets on the right side

We retrieved all currently existing SINA/SIAH amino acid sequences from the NCBI Refseq protein database by conducting separate BLAST searches using *Drosophila* SINA along with human SIAH1, SIAH2, and SIAH3 full-length proteins as the query sequences. The E-value cutoff was 10^−60^ for each of the four separate searches. A subset of 70 unique SINA/SIAH sequences, spanning the entire known taxonomy of the animal kingdom, was used for the phylogenetic analyses as shown in this study (Table 1 and Fig. 1). This collection contained SINA/SIAH sequences from 19 vertebrate species and 20 invertebrate species. Among the 19 vertebrate species, all four major classes of tetrapod vertebrates were represented, i.e. mammals (1–6), birds (7–8), reptiles (9–12), and amphibians (13) (these numeric numbers represent their positions as shown in Table 1). The three major groups of jawed fish were represented, including coelacanth (14), teleost fish (15–17), and cartilaginous fish (18). Additionally, lamprey (19) was utilized as a jawless vertebrate representative. Among the 20 invertebrate species, all major divisions of invertebrates were represented, including invertebrate deuterostomes (20–21), arthropods (22–25), nematodes (26–29), mollusks (30–31), annelids (32), platyhelminths (33), cnidarians (34–37), sponges (38), and placozoans (39). Using this manual selection of 70 sequences from 39 unique metazoan species, we constructed a master phylogenetic tree of the entire SINA/SIAH protein family (Fig. 1). Invertebrates and jawless vertebrates possess a single seven-in-absentia (*sina*) gene. Jawed vertebrates (gnathostomes) possess at least two SINA orthologs/homologs: *siah1* and *siah2* genes. The majority of gnathostomes possess three SINA orthologs/homologs: *siah1*, *siah2* and *siah3* genes within their genomes. Jawed vertebrate taxa for which BLAST searches did not retrieve a SIAH3 sequence include teleost fish and the squamate division of reptiles (includes lizards and snakes, excludes crocodiles and turtles). Thus, the loss of the *siah3* gene from these specific vertebrate lineages in gnathostomes appears to be a recent evolutionary event.

**Table 1:** List of sequences (*n* = 70) that were utilized in all evolutionary analyses as conducted in this study

	Taxonomy	Species	SIAH1	SIAH2	SIAH3
		Vertebrates			
1	Mammalia	*Homo sapiens*	NP_003022.3	NP_005058.3	NP_942146.2
2	Mammalia	*Pan troglodytes*	NP_001233288.1	XP_516819.2	XP_522672.3
3	Mammalia	*Mus musculus*	NP_033198.1	NP_033200.2	NP_001121565.1
4	Mammalia	*Bos taurus*	XP_005218736.1	NP_001193983.1	NP_001192350.1
5	Mammalia	*Tursiops truncatus*	XP_019788031.1	XP_019789908.1	XP_019792000.1
6	Mammalia	*Monodelphis domestica*	XP_007474893.1	XP_001363407.1	XP_007501643.1
7	Aves	*Gallus gallus*	XP_015147897.1	XP_426719.2	XP_417044.1
8	Aves	*Sturnus vulgaris*	XP_014738058.1	XP_014741604.1	XP_014750862.1
9	Reptilia	*Pogona vitticeps*	XP_020643987.1	XP_020637543.1	
10	Reptilia	*Thamnophis sirtalis*	XP_013913965.1	XP_013927960.1	
11	Reptilia	*Alligator mississippiensis*	XP_019352994.1	XP_006259223.1	XP_019356083.1
12	Reptilia	*Chrysemys picta*	XP_008165194.1	XP_005286979.1	XP_005287220.1
13	Amphibia	*Xenopus tropicalis*	NP_001015836.1	NP_001095281.1	XP_002941011.1
14	Sacropterygii	*Latimeria chalumnae*	XP_005998413.1	XP_006008925.1	XP_014352065.1
15	Neopterygii	*Danio rerio*	NP_955815.1	NP_956721.2	
16	Neopterygii	*Salmo salar*	XP_013979721.1	XP_014052924.1	
17	Neopterygii	*Oreochromis niloticus*	XP_019217515.1	XP_003459581.3	
18	Chondrichthyes	*Callorhinchus milii*	XP_007887616.1	XP_007889716.1	XP_007889930.1
19	Cyclostomata	*Petromyzon marinus*	S4R9G1_PETMA		
		Invertebrates	SINA		
20	Cephalochordata	*Branchiostoma floridae*	XP_002609562.1		
21	Echinodermata	*Strongylocentrotus purpuratus*	XP_797311.2		
22	Arthropoda	*Drosophila melanogaster*	NP_476725.1		
23	Arthropoda	*Anopheles gambiae*	XP_001688791.1		
24	Arthropoda	*Apis mellifera*	XP_394284.2		
25	Arthropoda	*Acyrthosiphon pisum*	XP_008186354.1		
26	Nematoda	*Caenorhabditis elegans*	NP_500409.1		
27	Nematoda	*Necator americanus*	XP_013306181.1		
28	Nematoda	*Brugia malayi*	XP_001898781.1		
29	Nematoda	*Trichinella spiralis*	XP_003379392.1		
30	Spiralia	*Octopus bimaculoides*	XP_014779248.1		
31	Spiralia	*Crassostrea gigas*	XP_011434753.1		
32	Spiralia	*Helobdella robusta*	XP_009028819.1		
33	Spiralia	*Schistosoma mansoni*	XP_018646300.1		
34	Cnidaria	*Nematostella vectensis*	XP_001637064.1		
35	Cnidaria	*Orbicella faveolata*	XP_020623250.1		
36	Cnidaria	*Acropora digitifera*	XP_015766642.1		
37	Cnidaria	*Hydra vulgaris*	XP_002162099.1		
38	Porifera	*Amphimedon queenslandica*	XP_019850617.1		
39	Placozoa	*Trichoplax adhaerens*	XP_002108034.1		

Taxonomic designation for representative species (*n* = 39) is listed to the left of its name. With the exception of the sequence from *Petromyzon marinus*, which was acquired from UniProtKB, all amino acid sequence identifiers presented in this table refer to their NCBI Genbank accession numbers

We utilized the maximum-likelihood method (ML) to construct the phylogenetic tree. The chosen model of sequence evolution was LG + G4 + F, and 100 bootstrap replicates were performed to assess the validity of the groupings within the generated ML tree (log likelihood = −9417.10). The sequences for *Amphimedon queenslandica* and *Trichoplax adhaerens* were utilized as the phylogenetic outgroup. This phylogenetic analysis does not resolve any ancestral evolutionary relationships among the three vertebrate SIAH subfamilies, nor does it recover them as a monophyletic grouping. There was confident support for the individual subtrees of vertebrate SIAH1 (94) and SIAH3 (99). By contrast, a considerably weaker bootstrap value (60) was obtained for monophyly of the SIAH2 subtree, and a value of similar magnitude (59) was obtained for a sister-group relationship between the SIAH2 and SIAH3 sequences (Fig. 1). Among the three vertebrate SIAH proteins, the SIAH3 subfamily exhibited the highest rate of amino acid substitution (measured in number of substitutions per site). These values were determined by summing branch lengths along the path starting from the node adjacent to the subtree’s root and ending at the tip of the *H. sapiens* branch within each subtree (designated “root-to-tip distance”). The root-to-tip distance for SIAH3 subtree is the greatest by a large margin (2.58 substitutions/site). The SIAH2 subfamily, with a root-to-tip distance of 0.15 substitutions/site, exhibits a mutation rate that is over 2-fold higher than that of the SIAH1 subfamily (0.07 substitutions/site). Moreover, SIAH3’s mutational rate is 17-fold higher across the vertebrate lineage when compared with SIAH2’s mutational rate, and 34-fold higher when compared with SIAH1’s mutational rate.

As the first identified member of this evolutionarily conserved family of RING E3 ligases, *Drosophila* SINA shares an extensive degree of amino acid sequence identity/similarity to vertebrate SIAH1, SIAH2 and SIAH3 proteins [14, 21]. The phylogenetic analyses presented here demonstrate that vertebrate SIAH1, SIAH2, and SIAH3 are all equally orthologous to invertebrate SINA (Fig. 1). There are two possible scenarios for the emergence of the three SIAH paralogs from an ancestral SINA/SIAH gene that existed in the vertebrate last common ancestor (LCA): (1) tandem gene duplications or (2) successive whole genome duplications. The details of these *siah* gene duplication events are not yet fully understood, as the phylogenetic analysis presented here does not yield enough resolution (low node supports) to reveal their lineage relationships. Thus, SIAH1, SIAH2 and SIAH3 are three paralogous lineages in vertebrates, but their exact evolutionary history remains unclear.

Invertebrate SINA sequences have evidently undergone significant divergent evolution within their various lineages since the bifurcation of bilaterians into protostomes and deuterostomes. This is demonstrated by the relatively strong bootstrap values obtained for the Arthropoda (71), Nematoda (96), and Spiralia (91) subtrees (Fig. 1). The analysis also contained SINA sequences from Cnidaria, a clade that is a sister-group to the entire bilaterian lineage. Their overall placement within the tree topology was correct; however, the exclusion of *Hydra vulgaris* from the well-supported Cnidaria clade suggests a significant sequence dissimilarity among the hydrozoan and anthozoan branches of this lineage. The phylogenetic analysis presented in Fig. 1 also helped to resolve ambiguity regarding the classification of the sequence retrieved from *Petromyzon marinus*. It was previously suspected that this was a SINA protein since the *P. marinus* genome only possesses one sequence belonging to the SINA/SIAH combined family, much like the other invertebrates with a single SINA protein. However, the inclusion of *P. marinus* within the well-supported SIAH1 clade provides support for an alternative view. *Petromyzon marinus* contains a SIAH1 sequence with high similarity to SIAH1 of gnathostomes. There may have once been SIAH2 and SIAH3 genes in the genomes of ancestral jawless vertebrates that were subsequently lost over evolutionary time and are now absent from extant species.

The internal node supports within the vertebrate SIAH paralog subtrees also show great discrepancy. For the SIAH1 subtree, the only clade with some degree of bootstrap support is teleost fish (65), with all other SIAH1 sequences lumped into a sister-group to teleosts. SIAH2 sequences produce a bifurcating subtree with a correct tetrapod clade (79) and an incorrect grouping containing all fish SIAH2 sequences (84). The SIAH3 subtree exhibits the most robust recovery of an accurate vertebrate phylogeny. The *C. milii* and *L. chalumnae* branches are correctly placed in their basal position to tetrapods with maximal bootstrap support. The monophyletic tetrapod clade was recovered with a similar support value (81) as the SIAH2 tree. In contrast to the SIAH1 and SIAH2 trees, moderately strong support was obtained for a monophyletic mammalian clade (78) as well as a placental mammal group within this clade (73). Only the SIAH3 subfamily is congruent with the established phylogeny of vertebrate species as reported in the literature. The extraordinary amino acid sequence conservation in the vertebrate SIAH1 and SIAH2 subfamilies likely leads to a lack of demarcating phylogenetic signals, hampering the proper reconstruction of the expected species tree within the SIAH family.

#### Structural motifs and functional topology of the invertebrate SINA subfamily

**Fig. 2:**
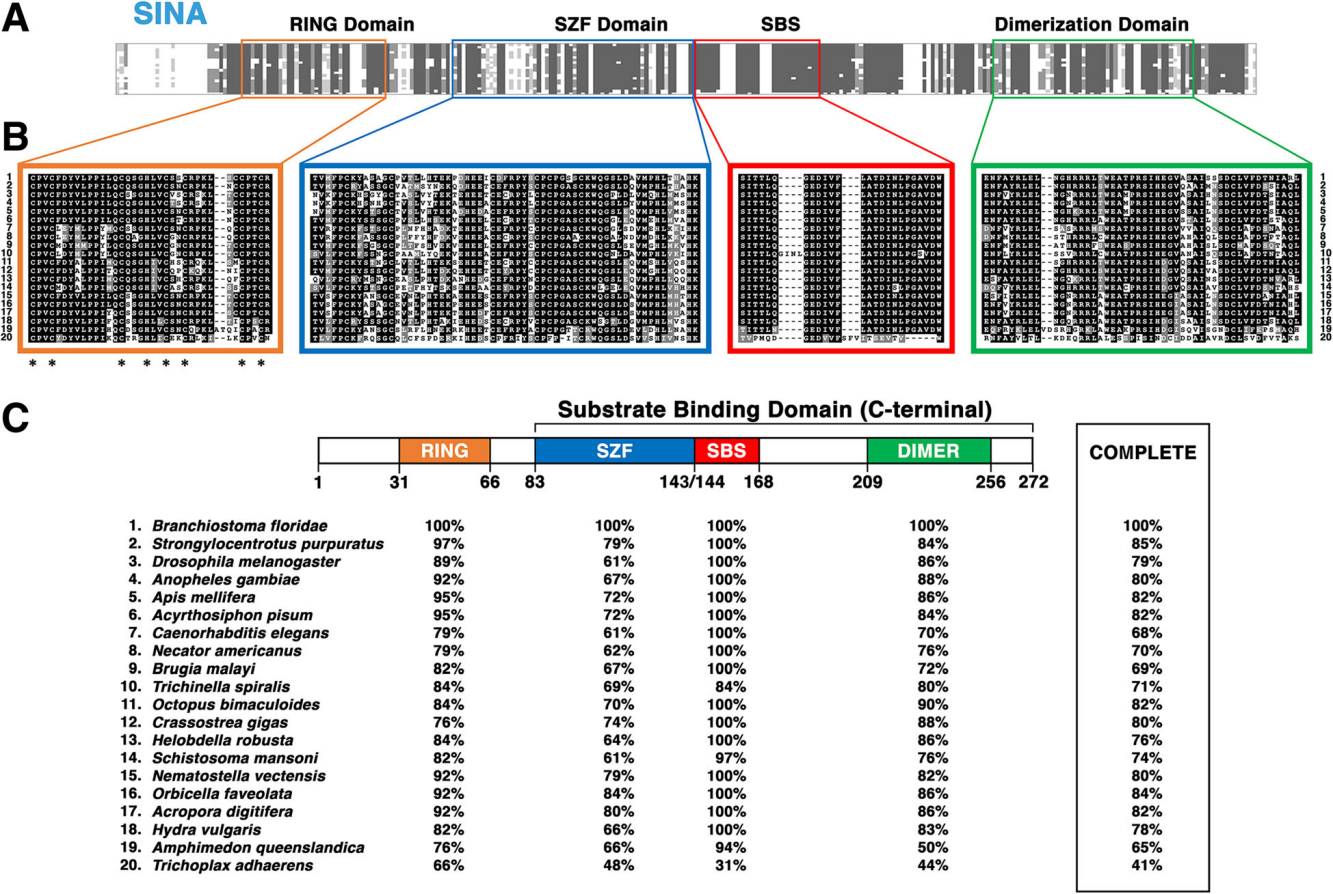
Sequence alignment of the invertebrate SINA subfamily reveals its invariant amino acid residues, and the four conserved structural motifs. Sequence comparison of SINA proteins from 20 representative invertebrate species (#1-#20) is shown. **a** Overview of the entire alignment produced by the 20 invertebrate SINA sequences. Four key functional domains are marked in four distinct colors: RING domain (*orange*), SZF domain (*blue*), SBS (*red*), and DIMER domain (*green*). **b** Schematic illustration of amino acid conservation within the 4 domains of the SINA sequences is shown. Amino acid identity is shown as white letters in a *black box*, amino acid similarity is shown as white letters in a *grey box*, and amino acid divergence is shown as black letters in a *white box*. The asterisks located below the RING domain alignment indicate unanimous conservation of the cysteine/histidine zinc-binding residues. **c** The percentages of amino acid conservation in each distinct domain and the entire SINA sequence between *Branchiostoma floridae* and each of the representative invertebrate species are shown. The diagram of the domain architecture was based on *B. floridae* SINA

To identify the conserved amino acid residues and structural motifs of the invertebrate SINA family, we conducted a functional domain analysis from 20 invertebrate SINA sequences as selected in this study (Fig. 2). A very noticeable observation upon obtaining the raw alignment is the high degree of the length variance in the highly diverse and variable N-terminal sequences of invertebrate SINAs. For invertebrate SINA proteins, the general length of the evolutionarily conserved C-terminal SINA sequence (comprised of the 4 known functional domains, RING, SZF, SBS and DIMER) was fairly consistent among the 20 diverse invertebrate species in our analysis (Fig. 2a and b). By contrast, the length of the N-terminal sequence preceding the conserved RING domain in invertebrate SINA sequences was quite variable and diverse, ranging anywhere between 26 and 155 amino acids in length (see Additional file 1).

For the purposes of standardizing and refining the invertebrate SINA sequencing alignment, *Branchiostoma floridae* (a basal chordate with ≥ 90% identity to human SIAH1 in all functional domains except SZF) was chosen as a reference sequence, and all positions that resulted in gaps within this sequence exclusive of the four functional domains were eliminated from the refined alignment (Fig. 2). Sequence alignments indicated a considerable degree of amino acid diversity in the RING, SZF, and DIMER domains of invertebrate SINA sequences (Fig. 2). The SBS exhibits a higher level of conservation, but also possesses noticeable species-specific insertions that produce gaps in the alignment (Fig. 2a and b). Quantification of the percent similarity in invertebrate SINA amino acid sequences as compared to the *B. floridae* reference SINA sequence support this observation (Fig. 2c). Several invertebrates share 100% amino acid identity with the SBS domain of *B. floridae*; however, there are notably reduced amino acid identities observed within the RING, SZF, and DIMER domains of this SINA family (Fig. 2c). Quantification of the two outgroup sequences indicates that *Trichoplax adhaerens* SINA completely lacks the evolutionary conservation observed in all other metazoan SINA/SIAH sequences. It is essentially a unique “outlier” sequence within the family, as evidenced by the fact that its SBS shares just 31% identity with the highly conserved SBS motif found in SINA, SIAH1, and SIAH2 sequences (Fig. 2c).

#### Structural motifs and functional topology of the vertebrate SIAH1 subfamily

**Fig. 3:**
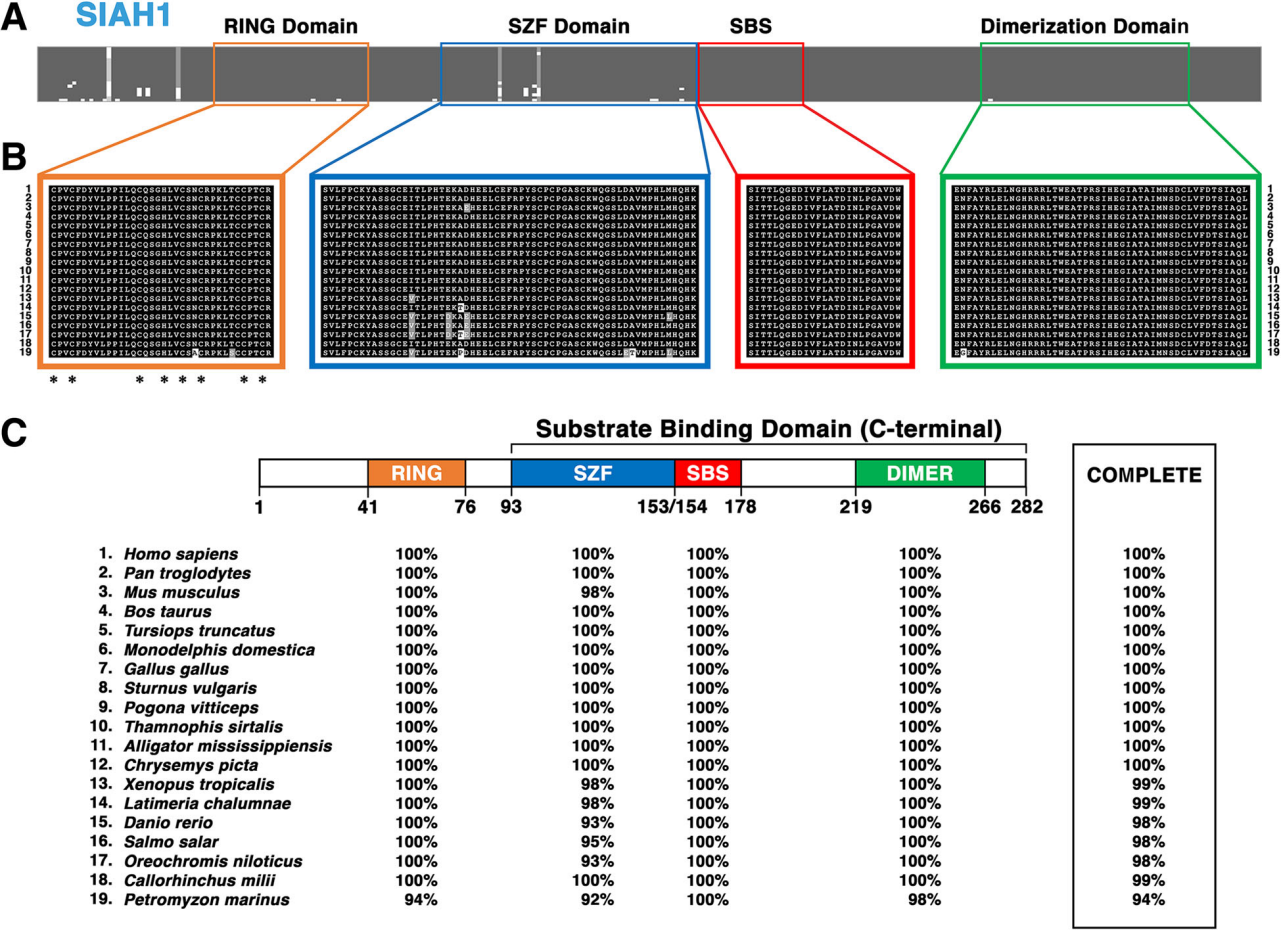
Sequence alignment of the vertebrate SIAH1 subfamily reveals its invariant amino acid residues, and the four conserved structural motifs. Sequence comparison of SIAH1 proteins from 19 representative vertebrate species (#1-#19) is shown. **a** The level of amino acid conservation in the N-terminal portion (#1 to #40) is high among SIAH1 sequences. Four key functional domains are marked in four distinct colors: RING domain (*orange*), SZF domain (*blue*), SBS (*red*), and DIMER domain (*green*). **b** Schematic illustration of amino acid conservation within the 4 domains of the SIAH1 sequences is shown. Amino acid identity is shown as white letters in a *black box*, amino acid similarity is shown as white letters in a *grey box*, and amino acid divergence is shown as black letters in a *white box*. The asterisks located below the RING domain alignment indicate unanimous conservation of the cysteine (Cys)/histidine (His) zinc-binding residues. **c** The percentages of amino acid conservation in each distinct domain and the entire SIAH1 sequence between human and each of the representative vertebrate species are shown. The diagram of the domain architecture was based on *Homo sapiens* SIAH1

Here, we focused on identifying the invariant amino acid residues and highly conserved structural motifs among the SIAH1 subfamily in all vertebrate species (Fig. 3). The alignment of the vertebrate SIAH1 sequences demonstrates an extraordinary degree of amino acid sequence identity among SIAH1 orthologs (Fig. 3a), even within an N-terminal sequence that was quite divergent in the invertebrate SINA family (Fig. 2). The RING and SZF domains in the SIAH1 subfamily possess 8 immutable zinc-coordinating histidine (His) and cysteine (Cys) amino acid residues [39–41]. In fact, all aligned gnathostome SIAH1 sequences possess 100% sequence identity to each other within their RING domains (Fig. 3b and c). The SIAH1 sequence for *Petromyzon marinus* only possesses two amino acid differences from this conserved RING domain sequence. Similarly, the SIAH1 sequences from jawed vertebrates demonstrated 100% interspecies conservation within the SBS and DIMER domains (Fig. 3b and c). *Petromyzon marinus* SIAH1 is also identical to the vertebrate SIAH1 SBS domain, and only bears a single divergent amino acid compared to the DIMER domain sequence. The SZF contains the lowest degree of amino acid conservation out of the four domains, with *P. marinus* having 92% identity with human SIAH1 (Fig. 3b and c). Additionally, it is the only functional domain to exhibit any degree of amino acid divergence amongst all the gnathostome SIAH1 sequences (Fig. 3c). Together, this data shows that SIAH1 sequences have maintained an extraordinarily high degree of amino acid conservation ever since the vertebrate SIAH1 paralog first originated from the vertebrate LCA’s SIAH protein (Fig. 3).

#### Structural motifs and functional topology of the vertebrate SIAH2 subfamily

**Fig. 4:**
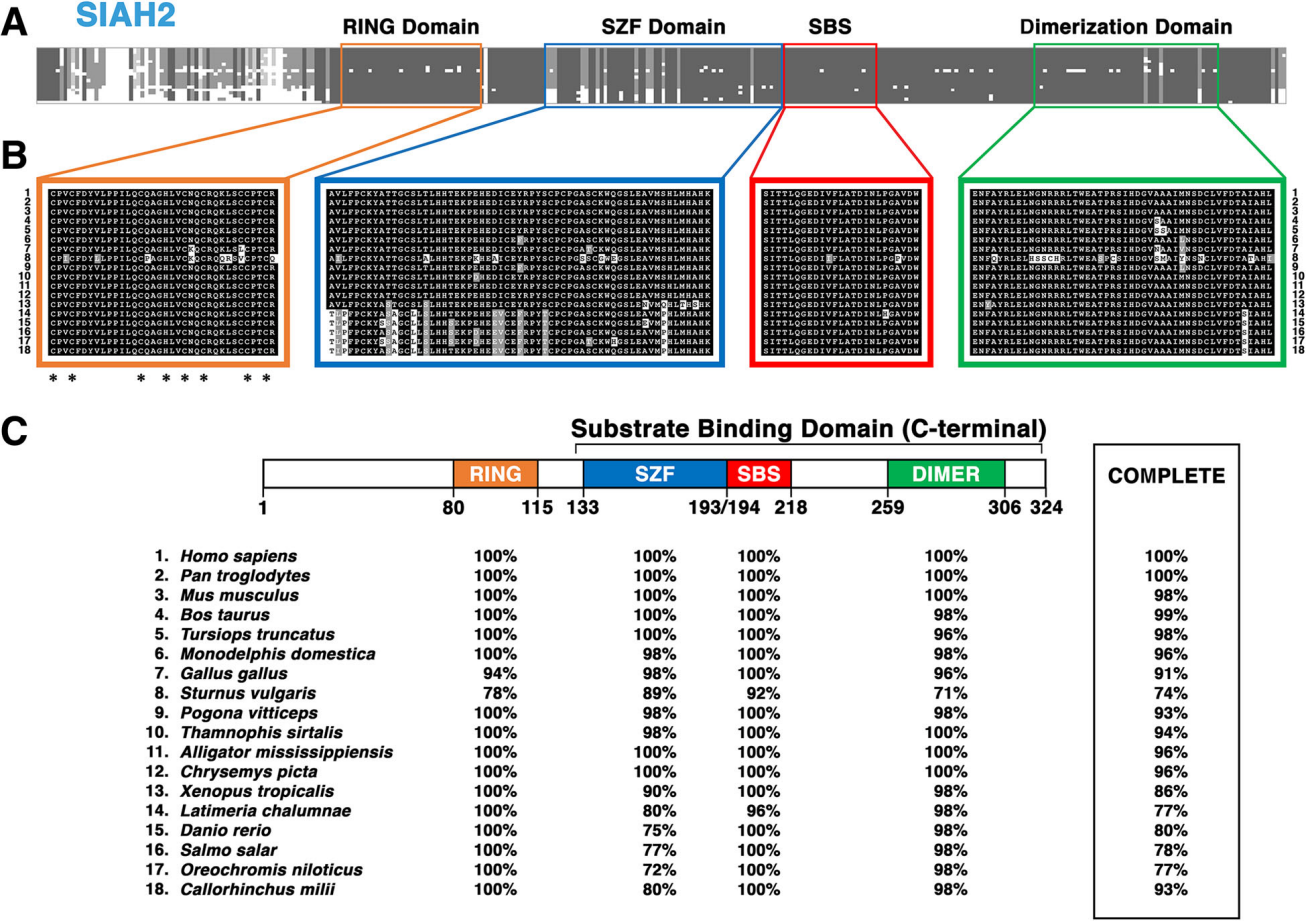
Sequence alignment of the vertebrate SIAH2 subfamily reveals its invariant amino acid residues, and the four conserved structural motifs. Sequence comparison of SIAH2 proteins from 18 representative vertebrate species (#1-#18) is shown. **a** The level of amino acid divergence in the N-terminal fragments (#1 to #80) is high. Four key functional domains are marked in four distinct colors: RING domain (*orange*), SZF domain (*blue*), SBS (*red*), and DIMER domain (*green*). **b** Schematic illustration of amino acid conservation within the 4 domains of the SIAH2 sequences is shown. Amino acid identity is shown as white letters in a *black box*, amino acid similarity is shown as white letters in a *grey box*, and amino acid divergence is shown as black letters in a *white box*. The asterisks located below the RING domain alignment indicate unanimous conservation of the cysteine (Cys)/histidine (His) zinc-binding residues. **c** The percentages of amino acid conservation in each distinct domain and the entire SIAH2 sequence between human and each of the representative vertebrate species are shown. The diagram of the domain architecture was based on *Homo sapiens* SIAH2. The SIAH2 sequence for *C. milii* was incomplete, and these gaps induced by the incompleteness of the sequence were disregarded when calculating conservation across the whole protein

To identify the invariant amino acid residues and highly conserved structural motifs in the SIAH2 subfamily, we aligned vertebrate SIAH2 sequences from 18 diverse species of jawed vertebrates. The sampling of these species used in this analysis of the SIAH2 subtree includes representatives from all the major taxa within the gnathostome clade, with an emphasis on mammals (Fig. 4). Vertebrate SIAH2 has an extended N-terminal fragment that is 40 amino acids longer than that of SIAH1 (Fig. 4). The unique 80 amino acid N-terminal fragments in the SIAH2 subfamily are quite diverse, while the SIAH2 core sequences (#80-#324) share a high level of amino acid identity with the SIAH1 core sequence (#41-#282) (Fig. 4). Like SIAH1 orthologs, SIAH2 orthologs have 4 essential functional domains: the RING, SZF, SBD, and DIMER domains (Fig. 4a and b). The evolutionary conservation in the SIAH2 subfamily is illustrated by the extraordinarily high level of amino acid identities observed in all four functional domains (Fig. 4).

Sequence homology analysis of the SIAH2 subfamily shows that the four distinct SIAH2 functional domains exhibit a high degree of evolutionary conservation (Fig. 4b and c). Like SIAH1 orthologs, the SBS motif exhibits the highest degree of conservation in these SIAH2 orthologs (Fig. 4b). Sixteen of the eighteen metazoan SIAH2 SBS sequences are 100% identical to the human SIAH2 SBS, with two species (*S. vulgaris,* and *L. chalumnae*) evolving only two and one amino acid substitutions, respectively. The extraordinarily high level of amino acid conservation was also observed throughout the RING (100%), SZF (98%) and DIMER (94%) domains of vertebrate SIAH2 orthologs (Fig. 4b and c, and Table 2). When comparing SIAH2 amino acid sequences from all gnathostomes, the SIAH2 sequence conservation observed in the SBS domain is significantly higher compared to that of the other three domains (Fig. 4). For example, 22/25 (88%) of amino acid residues in the SBS are identical among all 18 vertebrate SIAH2 sequences as aligned (Fig. 4). In contrast, just 32/48 (67%) of amino acid residues in the DIMER domain are unanimously conserved (Fig. 4c). The RING domains share 67% identity and the SZF domains share 64% identity (Fig. 4b and c, and Table 2). Similar to vertebrate SIAH1, all eight metal-coordinating cysteine (Cys) and histidine (His) residues are immutable amino acids in both the RING and SZF domains of the SIAH2 subfamily (Fig. 4b). Interestingly, the majority of the divergent amino acids in the domains belong to *Sturnus vulgaris*, suggesting that there has been accelerated SIAH2 amino acid sequence evolution in birds of the Passeriformes family in comparison to other tetrapod species.

**Table 2:** Comparison of amino acid identities observed in the three SIAH paralogs

Consensus sequence	RING	SZF	SBS	DIMER	WHOLE
SINA	100%	100%	100%	100%	100%
SIAH1	100%	89%	100%	90%	88%
SIAH2	89%	83%	100%	85%	72%
SIAH3	28%	25%	60%	63%	45%

Percentage identity values for each paralog’s domains were calculated relative to the consensus sequence of invertebrate SINA, the mutual ortholog of all three SIAH paralogs

#### Structural motifs and functional topology of the vertebrate SIAH3 subfamily

**Fig. 5:**
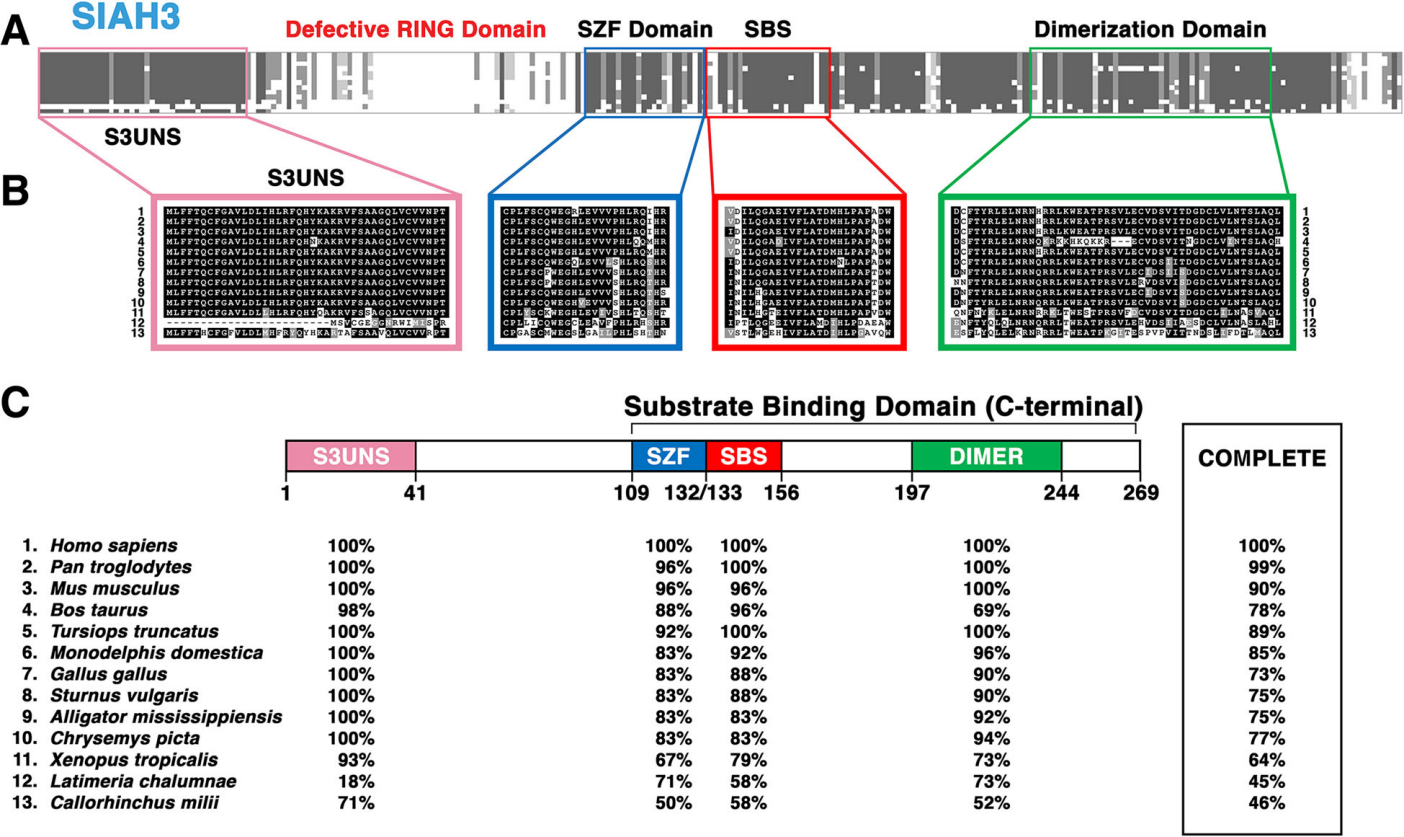
Sequence alignment of the vertebrate SIAH3 subfamily reveals its invariant amino acid residues, absence of RING domain, and conserved structural motifs. Sequence comparison of SIAH3 proteins from 13 representative vertebrate species (#1-#13) is shown. **a** The level of amino acid conservation in the N-terminal fragments is high, while the portion of the protein sequences that would be expected to contain the RING domain is highly divergent compared to SIAH1 and SIAH2. Four key functional domains are marked in four distinct colors: SIAH3 unique N-terminal sequence (S3UNS, *pink*), SZF domain (*blue*), SBS (*red*) and DIMER domain (*green*). **b** Schematic illustration of amino acid conservation within the 4 distinct domains of the SIAH3 sequences is shown. Amino acid identity is shown as white letters in a *black box*, amino acid similarity is shown as white letters in a *grey box*, and amino acid divergence is shown as black letters in a *white box* . **c** The percentages of amino acid conservation in each distinct domain and the entire SIAH3 sequence between human and each of the representative vertebrate species are shown. The diagram of the domain architecture was based on *Homo sapiens* SIAH3

Vertebrate SIAH3 is the most divergent member of the vertebrate SIAH family E3 ligases. Unlike its vertebrate SIAH1 and SIAH2 counterparts, SIAH3 orthologs do not possess a functional RING domain, suggesting that SIAH3 is an enzymatically-inactive E3 ligase (Fig. 5a and b). In addition to the loss of the RING domain, SIAH3 contains only a single Zinc finger in the SZF domain compared to the double Zinc finger motif found in vertebrate SIAH1 and SIAH2 [23]. SIAH3 orthologs, apart from *Latimeria chalumnae,* possess SIAH 3-unique N-terminal sequences (S3UNS) that are highly conserved and evolutionarily unique to the SIAH3 subfamily, but are completely absent from the SIAH1 and SIAH2 subfamilies (Fig. 5). SIAH3 has 4 distinct functional motifs, including S3UNS, SZF, SBS and DIMER domains (Fig. 5a–c). Among the evolutionarily conserved SZF, SBS and DIMER domain in vertebrates, the SIAH3 subfamily exhibits a higher mutation rate and additional amino acid divergence when compared to SIAH1 and SIAH2 subfamilies (Fig. 5c).

#### Comparison of SINA, SIAH1, SIAH2 and SIAH3 consensus amino acid sequences in metazoans

**Fig. 6:**
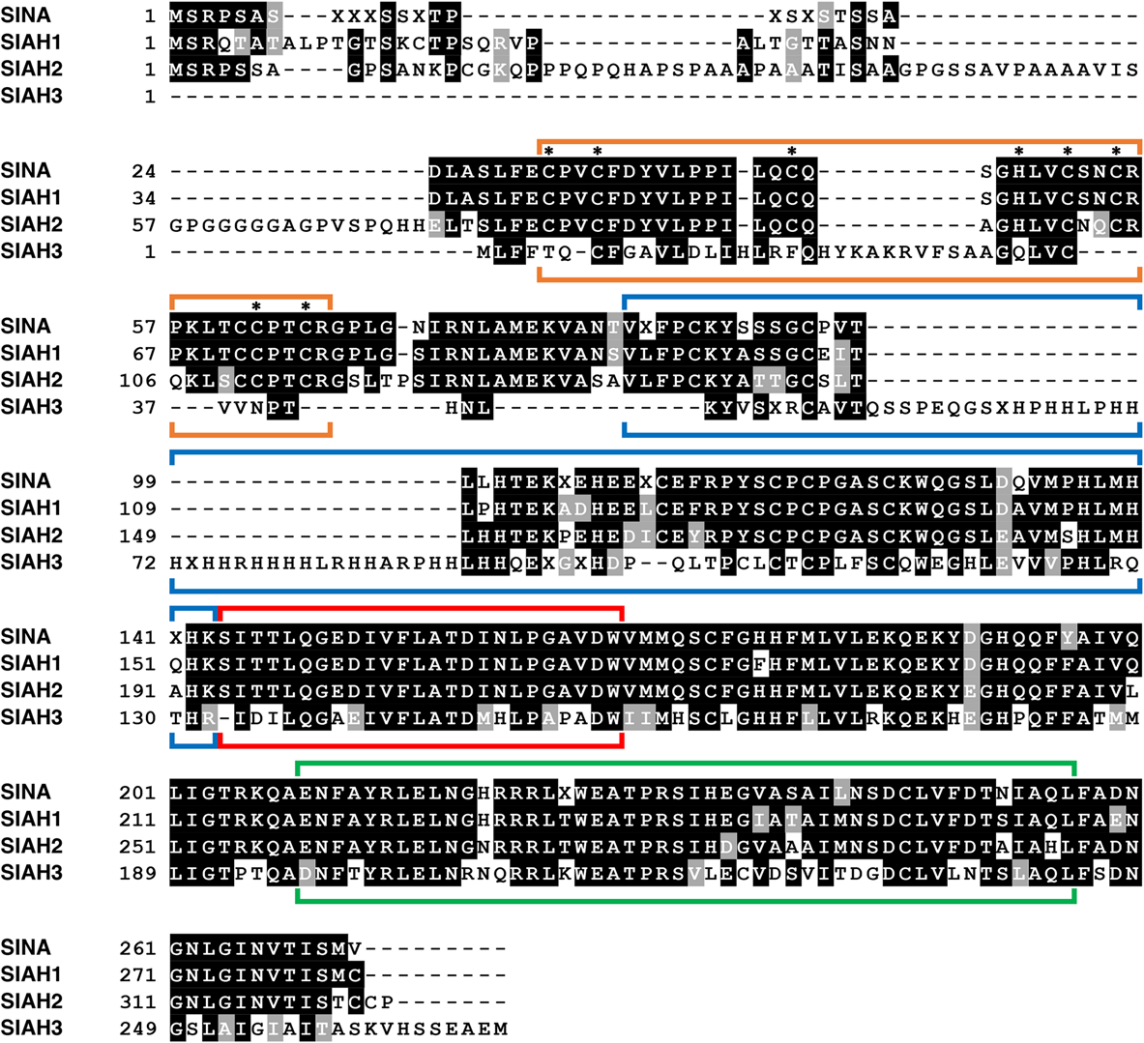
The consensus sequences of SINA, SIAH1, SIAH2, and SIAH3 were aligned to identify the invariant and divergent amino acid residues in this evolutionarily highly conserved SINA/SIAH E3 ligase family. There is a high level of amino acid conservation in the SBD domain in the SINA, SIAH1, SIAH2, and SIAH3 core consensus sequences in the SIAH family. The RING domain is marked by an *orange bracket*, the SZF domain by a *blue bracket*, the SBS by a *red bracket*, and the DIMER domain by a *green bracket*. Asterisks within the RING domain indicate the position of the invariant cysteine (Cys)/histidine (His) residues in SINA, SIAH1 and SIAH2. Amino acid positions marked with an “X” (instead of a valid one-letter amino acid abbreviation) indicate that the consensus at this site could not be resolved unambiguously. SINA, SIAH1, and SIAH2 share extensive sequence homology between each other in their core consensus sequences, whereas SIAH3 shows dramatic sequence divergence in the corresponding RING and SZF domains to those of SINA, SIAH1, and SIAH2 proteins. SINA, SIAH1, SIAH2, and SIAH3 share high levels of amino acid conservation in the SBS domains

To identify the invariant amino acid residues and conserved functional domain sequences in the SINA/SIAH family, we compared the core consensus sequences of invertebrate SINA with its three vertebrate SIAH orthologs: SIAH1, SIAH2 and SIAH3. We aligned the 4 core consensus sequences of full-length SINA, SIAH1, SIAH2 and SIAH3 extracted from Figs. 2, 3, 4 and 5 together (Fig. 6 and Table 2). SIAH1 and SIAH2 are two functional E3 ligases with high amino acid identity/similarity observed in their core consensus sequences, while SIAH3 is a nonfunctional and inactive E3 ligase that is missing a catalytically active RING domain (Fig. 5). The invertebrate SINA consensus sequence bears the highest degree of similarity to vertebrate SIAH1, as the majority of amino acids within the functional domains of these two consensus sequences are conserved (Table 2). Despite the conservation of these core consensus sequences, invertebrate SINA is still considered equally orthologous to each of the three vertebrate SIAH proteins. These orthologous relationships exist because all extant SIAH paralogs originated from duplication events involving the ancestral vertebrate SIAH protein. This ancestral vertebrate SIAH protein diverged from invertebrate SINA via a speciation event (the emergence of the vertebrate lineage). The SBS and DIMER domains are fully intact in all three SIAH paralogs, and exhibit the highest degree of amino acid conservation among the entire vertebrate SIAH family (Fig. 6). By contrast, SIAH3 lacks the high amino acid identity in the RING and SZF domains as reported in SIAH1 and SIAH2 proteins (Figs. 5 and 6). Additionally, the SIAH3 consensus sequence contains a histidine-rich region within the first half of the SZF observed in SIAH1 and SIAH2 consensus sequences (Figs. 5 and 6). Our phylogenetic analysis did not support the previous conclusion that the *siah3* gene is derived from a duplication of the *siah2* gene [23].

#### SIAH1, SIAH2 and SIAH3 mRNA expression in human cancer cells

**Fig. 7:**
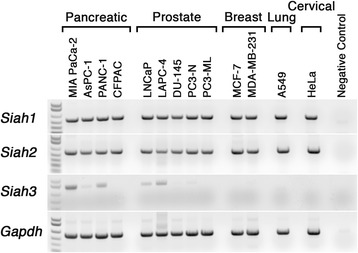
SIAH1, SIAH2 and SIAH3 mRNA expression in human cancer cell lines. Semi-quantitative reverse transcription – polymerase chain reaction (RT-PCR) analysis of *siah1*, *siah2* and *siah3* mRNA transcript expression in human cancer cell lines is shown. The relative expression levels of *siah1*, *siah2* and *siah2* mRNA transcripts in 13 epithelial cancer cells including pancreatic cancer cells (MiaPaCa, AsPC-1, PANC-1 and CFPAC), prostate tumor cell lines (LNCaP, LAPC-4. PC-3-N (normal), PC-3-ML (metastatic)), breast tumor cell lines (MCF-7 and MDA-MB-231), non-small cell lung cancer cell line (A549) and cervical cancer cell line (HeLa) were estimated semi-quantitatively for serial dilutions of the complementary DNA templates. Glyceraldehyde-3-phosphate dehydrogenase (GAPDH) mRNA transcript was used as an internal control. The RT-PCR mixture without cDNA template was used as a negative control

RT-PCR was performed to examine the expression levels of *siah1*, *siah2* and *siah3* mRNA transcripts in 15 human epithelial cell lines with a range of tumorigenicities (Fig. 7). These cell lines include human pancreatic, prostate, breast, lung and cervical cancer cell lines. Specific and unique PCR primers were used to synthesize mRNA products from each distinct SIAH subfamily. Our findings show that *siah1* and *siah2* mRNA transcripts are universally expressed in all human epithelial cell lines examined so far [36, 37], whereas *siah3* mRNA transcript is expressed in a small subset of human tumor cell lines. This result suggests a biological function of SIAH3 that is distinct from those of SIAH1 and SIAH2 in human tumor biology (Fig. 7).

## Discussion

The field of cancer biology desperately needs a more effective method for controlling and conquering oncogenic K-RAS hyperactivation in metastatic human cancer. With our new strategy of targeting SINA/SIAH1/SIAH2, the most conserved and the most downstream “signaling gatekeeper” identified thus far in the RAS signaling pathway, we aim to bypass obstacles such as the extensive bifurcation, cross-talk and dynamic feedback controls downstream of several major compensatory K-RAS effector pathways to impede and block oncogenic K-RAS-driven malignant tumors [10]. To determine the biological function and evolutionary constraints of the SINA/SIAH family of E3 ligases, we conducted a phylogenetic analysis to identify the invariant amino acids and conserved functional motifs in the SINA/SIAH family among all major taxa of metazoans. The co-existence of the three unique SINA orthologs, SIAH1, SIAH2, and SIAH3, may represent an interesting event of genome evolution, gene duplication, and gene divergence in vertebrates. The three vertebrate SIAH paralogs were not recovered as a monophyletic grouping, which could be a result of long-branch attraction between the Cnidaria SINA sequences and vertebrate SIAH1 sequences. Given the high amino acid identity observed in one-to-one alignments of human SIAH1 with cnidarian SINA sequences, this possibility may be a logical deduction. Future phylogenetic analyses with greater taxon sampling and density, combined with dN/dS calculations on the SINA/SIAH protein-coding nucleotide sequences to detect residues under positive selection, will be required. They will help to further elucidate the overall evolutionary relationship between the three vertebrate SIAH paralogs and where they fit within the context of the metazoan evolution of this medically important family of RING-domain E3 ligases in RAS signal transduction in all metazoan cells.

The vertebrate SIAH1 and SIAH2 core consensus sequences exhibit the highest degree of amino acid identities within this RING domain E3 ligase family (Fig. 6). The invertebrate SINA underwent significant divergent evolution in the protostome lineage after the divergence of bilaterians (Fig. 1). Unlike vertebrate SIAH1 and SIAH2 orthologs, we report that SIAH3 orthologs exhibit the highest rates of amino acid substitution and sequence divergence within the gnathostome lineage. Based on phylogenetic analyses and amino acid substitution patterns, we speculate that the emergence of this SIAH3 subfamily, unique to jawed vertebrates, may have suppressor effects on its sibling SIAH1 and SIAH2 subfamilies. This finding may have possible medical relevance in cancer biology. *Siah3* mRNA transcript expression in several human cancer cell lines as shown in Fig. 7, raised the interesting possibility that nonfunctional SIAH3 may function as an endogenous SIAH inhibitor that regulates and antagonizes SIAH1 and SIAH2 biological activity through its DIMER domain to suppress and inhibit SIAH1 and SIAH2 biological activities to control cell proliferation, tissue growth, pattern formation, and homeostasis. This idea is supported by several previous studies that demonstrated that the RING domain-deleted SIAH1 or SIAH2 mutants (termed SIAH1/2 dominant-negative) functionally ablated endogenous activity of SIAH1 and SIAH2 in cancer biology [23, 27, 32, 38, 42, 43].

The molecular phylogenetic analysis of the SINA/SIAH family provides valuable insights into immutable amino acid residues and conserved functional motifs across the entire metazoan kingdom. Previous biomedical studies have narrowly focused on SINA, SIAH1, and SIAH2 in *Drosophila*, mice, and humans [14, 21, 32, 44–47]. Based on the phylogenetic analyses in metazoans, we suggest that SIAH3 is a new member of the SINA/SIAH E3 ligase family that lacks functional E3 ligase activity. The phylogenetic analysis conducted in this study provides a new framework and a novel evolutionary perspective with which we can identify and dissect the invariant and divergent amino acid residues and the conserved functional domains of SINA/SIAH proteins at the molecular level for phylomedicine. This is especially useful in the context of the SINA/SIAH1/2 gatekeeper function required for proper K-RAS activation and context-dependent RAS signal transmission [10, 36, 37]. This phylogenetic analysis of SINA/SIAH evolution in the animal kingdom is likely to provide valuable insights into the logical design of effective anti-K-RAS drugs that selectively target and specifically inhibit human SIAH1/2 proteins to rapidly shut down oncogenic K-RAS-driven malignant tumor growth and block metastatic cancer cell dissemination.

The discovery of SIAH3’s increased expression in a subset of human cancer cells presents an interesting opportunity for novel drug discovery. By taking advantage of nonfunctional SIAH3 as a putative endogenous SIAH inhibitor, we may be able to develop a novel anti-SIAH1 and anti-SIAH2 strategy by utilizing SIAH3 expression to antagonize oncogenic K-RAS-driven metastatic human cancer cells. SIAH3 shares a common ancestry with SIAH1 and SIAH2, as well as three conserved structural motifs (i.e., SBS, SZF and DIMER domains). It is conceivable that SIAH3 may function as a highly specific inhibitor of endogenous SIAH1 and SIAH2 activity by binding to SIAH1/2 via its DIMER domain. Additional work will be conducted to examine the interplay between SIAH3 and SIAH1/SIAH2 in oncogenic K-RAS-driven human cancers in the future. By focusing on these invariant amino acid residues and conserved functional motifs identified in the SINA/SIAH superfamily, we aim to design a phylogenetic-based, targeted and more specific anti-SIAH-based anticancer strategy to both impede and eradicate oncogenic K-RAS-driven metastatic human cancers for clinical translation in the future.

## Conclusions

This study demonstrates the extraordinarily high degree of evolutionary conservation in the SINA/SIAH family of E3 ligases in metazoans. SINA/SIAH proteins evidently originated early in metazoan evolution. The phylogenetic analysis presented here indicates that invertebrate SINA is a mutual ortholog of the three vertebrate SIAH paralogs, and future analyses will help resolve the exact evolutionary lineage of this unique RING-domain E3 ligase family. These ancestral SINA/SIAH E3 ligases occur under stringent evolutionary selection pressure that prevents diversification of their core sequences in all major metazoan taxonomy groups, as shown in the highly conserved SBS domain, as well as all immutable Cys/His zinc-binding residues within the RING and SZF domains of SIAH1, SIAH2, and SINA proteins. SIAH3 orthologs lack the conserved Zinc-binding Cys/His residues, suggesting a loss of the functional E3 ligase activity. Together, the phylogenetic analysis of the SINA/SIAH family can be utilized to pinpoint the invariable amino acid residues and conserved structural domains that are absolutely critical for their enzymatic functions and biological activities in transmitting active RAS signal in metazoa. By analyzing the evolutionary relationship between invertebrate SINA and its vertebrate SIAH paralogs (SIAH1, SIAH2, and SIAH3), we have gained an in-depth understanding of the extraordinarily high degree of amino acid conservation in this medically significant gene family. This knowledge will promote a phylogenetic-based SIAH-centered drug design toward generating useful SIAH-specific peptides and SIAH small molecule inhibitors as new and more efficacious therapeutics to eradicate oncogenic K-RAS-driven metastatic cancer in the future.

## Methods

### Sequence database search and data partitioning

The putative vertebrate paralogs of human SIAH1 were determined using the Ensembl gene tree associated with the protein (GeneTree ENSGT00390000005434). PSI-BLAST searches were performed on the NCBI protein database using the three SIAH paralogs (SIAH1, SIAH2 and SIAH3) from *Homo sapiens* and SINA from *Drosophila melanogaster* as the query sequences. The default algorithm parameters were utilized for each query, except for Max target sequences (1000) and the E-value threshold (10^−60^). BLAST hits returned from the SIAH1 and SIAH2 searches were included in the master sequence collection. Table 1 is a subset of the master sequence collection if (a) they were >240 amino acids in length, and (b) produced an alignment with the functional domain-containing region of their human orthologs. The same length criteria were applied for hits from the SINA search, and the *Drosophila* query sequence was used as the reference for evaluating whether each hit produced a functional domain region alignment. In the case of the SIAH3 search, the length criteria and alignment-based selection filter were relaxed, and BLAST hits were included for species which returned results >200 amino acids in length.

A total of 70 sequences (20 SINA sequences from invertebrates; and 50 vertebrate SIAH sequences (19 SIAH1, 18 SIAH2, and 13 SIAH3 sequences respectively) were manually selected from the master collection of BLAST hits across all four searches for utilization in the phylogenetic analysis (“Main dataset”) (Table 1 and Additional file 2: Table S1). The sequence selection was targeted so that a balanced sampling of vertebrate and invertebrate species, in addition to adequate representation of the major metazoan taxonomy groupings, was achieved. The *Petromyzon marinus* SIAH1 sequence was manually downloaded from UniProtKB and added to the main dataset to obtain a total of 70 SINA/SIAH family sequences. For each vertebrate species within the Main dataset, all SIAH paralogs encoded by their genomes were included. The only vertebrate species within the Main dataset without three SIAH paralogs detected in their genomes by BLAST searches are the teleosts (*Danio rerio*, *Salmo salar*, *Oreochromis niloticus*), squamates (*Pogona vitticeps*, *Thamnophis sirtalis*), and the jawless vertebrate representative *Petromyzon marinus* (Table 1).

To conduct subsequent functional domain analyses, the Main dataset was subdivided into four smaller datasets. A SINA dataset consisting of all 20 invertebrate SINA sequences was created, as well as three distinct datasets for each vertebrate SIAH paralog. The SIAH1, SIAH2, and SIAH3 datasets consisted of 19, 18, and 13 sequences, respectively (Table 1 and Additional file 2: Table S1).

### Construction of multiple sequence alignments

Protein sequences were aligned using the MAFFT algorithm in all instances. The SINA/SIAH family alignment utilized the Main dataset, resulting in an alignment of 70 sequences that was 534 positions in length (Additional file 3). To eliminate positions with less than 30% sequence coverage (i.e. gaps present in >70% of sequences), the alignment was trimmed to 305 positions for usage in phylogenetic analysis (Additional file 4).

Three individual alignments were also constructed for each vertebrate SIAH paralog using their respective datasets. Following completion of MAFFT alignment on each of these smaller datasets, each alignment was manually refined to eliminate positions that resulted in gaps within a designated “reference sequence”. In the case of each SIAH paralog alignment, the respective *Homo sapiens* amino acid sequence was chosen as the reference. The gap-trimming procedure caused reductions in alignment length as follows: 287 to 282 positions for SIAH1, 391 to 324 positions for SIAH2, 283 to 269 positions for SIAH3. It should be noted that the large majority of the trimmed positions across all three paralog alignments were within the N-terminal portion, and not the conserved functional domains identified in each distinct SIAH subtree.

Additionally, an alignment for the mutual SINA ortholog of the three vertebrate SIAH paralogs was built using the “SINA dataset”. For the SINA alignment, *Branchiostoma floridae* was chosen because it is considered a basal chordate. This taxonomic status puts it closer phylogenetically to vertebrates than any other species within the SINA dataset. The gap-trimming procedure was executed differently for the SINA alignment due to the presence of more significant gaps within the functional domain region. Instead of trimming gaps present within the entire reference sequence, the manual refinement procedure was restricted to the alignment’s N-terminal portion (i.e. all positions upstream of the *B. floridae* RING domain start that resulted in gaps were cut). Additionally, an overhang at the C-terminal that was only present in *C. elegans* was eliminated from the alignment. These procedures cut down the alignment length from 495 (original) to 287 (refined). The unprocessed, original alignments for all four proteins are available in Additional files 1, 5, 6 and 7, while the refined alignments used in the figures are contained in Additional files 8, 9, 10 and 11.

### Phylogenetic analysis of SINA/SIAH protein family

To select an optimal amino acid substitution model within the Maximum-likelihood (ML) framework, the model selection tool within MEGA7 was utilized. The refined SINA/SIAH family alignment was used as the input data. The output returned results from 56 models, and we narrowed our selection to 16 results from the general amino acid replacement matrices (Dayhoff, JTT, WAG, LG) which included a gamma parameter (+G) (see Additional file 12 for output spreadsheet). The LG + G + F model was selected for ML-based phylogenetic analysis.

All gathered SINA/SIAH sequences were aligned and subjected to phylogenetic reconstruction using MEGA7 software [48]. The MEGA7 analysis involved the refined family alignment (70 amino acid sequences with 305 positions) as the input data. The evolutionary history was inferred by using the Maximum Likelihood method based on the LG + G + F model, and a tree with log likelihood = −9417.10) was obtained. Bootstrapping with 100 replicates was applied as a test of phylogeny. Subtree-Pruning-Regrafting (SPR) level 5 was chosen as the ML heuristic method. Initial tree(s) for the heuristic search were obtained automatically by applying Neighbor-Join and BioNJ algorithms to a matrix of pairwise distances estimated using a JTT model, and then selecting the topology with superior log likelihood value. A discrete gamma distribution was used to model evolutionary rate differences among sites [4 categories (+G, parameter = 0.5852)]. The tree was drawn to scale, with branch lengths measured in the number of substitutions per site.

### Functional domain analysis

The locations and length of functional domains for all three human SIAH paralogs were derived from their respective entries in NCBI protein database. Using these domain sequences as the reference in each of the three individual paralog alignments, sub-alignments for each of the four domains were extracted. Sequence identity calculations for each domain were performed using the “Calculate distance matrix” function within UniPro UGENE. These values were reported as percentage identity (not excluding gaps), and were calculated using human SIAH1/SIAH2/SIAH3 sequence as the reference. For the SINA alignment, the *Branchiostoma floridae* sequence was utilized as the reference for calculating identity values.

### Generation and comparison of SINA/SIAH core consensus sequences

Consensus sequences were extracted and downloaded from the four individual sub-alignments using the default settings within UGENE. Any amino acid position that was ambiguous (i.e. two or more amino acids are prevalent with equal frequency at a given position) was replaced with an “X” character. The four resulting consensus sequences were aligned using MAFFT and the results of this alignment are presented in Additional file 13.

### RNA isolation, cDNA synthesis, and RT-PCR amplification of siah1, siah2, and siah3 mRNA transcripts in human cancer cell lines

Total RNA was isolated from cancer cell lines using RNeasy Mini Kit per the manufacturer’s instructions and extraction protocol (Qiagen. Germantown, MD). cDNA synthesis was carried out using AMV First Strand cDNA synthesis kit following the manufacturer’s protocol (New England BioLabs. Ipswich, MA). PCR amplification was performed using Expand High Fidelity PCR System (Roche. Indianapolis, IN). All primers were purchased from Integrated DNA Technologies (Coralville, IA).

The forward and reverse primers for PCR amplification of the *siah1* cDNA transcript were 5′-ATGAGCCGTCAGACTGCTACAG-3′ and 5′-CAGGACTGCATCATCACCCAGT-3′, respectively. The forward and reverse primers for PCR amplification of the *siah2* cDNA transcript were 5′-GCCATCGTCCTGCTCATTGGCA-3′ and 5′-ACCAATATGGGAAGGCAGGCAGGAAGGGGC-3′, respectively. The forward and reverse primers for PCR amplification of the *siah3* cDNA transcript were 5′-ATGCTTTTCTTTACCCAGTGCT-3′ and 5′-TCACATTTCAGCTTCTGAGGGGA-3′, respectively. The forward and reverse primers for PCR amplification of the *gapdh* cDNA transcript were 5′-AAAGGGTCATCATCTCTGCC-3′ and 5′-TGACAAAGTGGTCGTTGAGG-3′, respectively.

## Additional files

Additional file 1: Original alignment for SINA subtree. 20 invertebrate SINA sequences, no trimmed positions. (FASTA 11 kb)

Additional file 2: Table S1. Each SINA/SIAH sequence is linked with online accession by one click. List of SINA/SIAH protein sequences (*n* = 70) that were utilized in the phylogenetic analyses as presented in this study. Taxonomic designation for representative species (*n* = 39) is listed to the left of its name. With the exception of the sequence from *Petromyzon marinus*, which was acquired from UniProtKB, all amino acid sequence identifiers presented in this table refer to their NCBI Genbank accession numbers. Additional file 2: Table S1 is identical to Table 1, except that the online accession version of these SINA/SIAH sequences was included in the Additional file 2: Table S1. (DOCX 24 kb)

Additional file 3: Original alignment of entire SINA/SIAH database. 70 SINA/SIAH protein sequences, no trimmed positions. (FASTA 37 kb)

Additional file 4: Refined alignment of entire SINA/SIAH database. 70 SINA/SIAH sequences, trimmed according to Methods. (FASTA 21 kb)

Additional file 5: Original alignment for SIAH1 subtree. 19 vertebrate SIAH1 sequences, no trimmed positions. (FASTA 6 kb)

Additional file 6: Original alignment for SIAH2 subfamily. 18 vertebrate SIAH2 sequences, no trimmed positions. (FASTA 8 kb)

Additional file 7: Original alignment for SIAH3 subtree. 13 vertebrate SIAH3 sequences, no trimmed positions. (FASTA 4 kb)

Additional file 8: Refined alignment for SIAH1 subtree. 19 vertebrate SIAH1 sequences, trimmed according to Methods. (FASTA 6 kb)

Additional file 9: Refined alignment for SIAH2 subtree. 18 vertebrate SIAH2 sequences, trimmed according to Methods. (FASTA 7 kb)

Additional file 10: Refined alignment for SIAH3 subtree. 13 vertebrate SIAH3 sequences, trimmed according to Methods. (FASTA 4 kb)

Additional file 11: Refined alignment for SINA subtree. 20 invertebrate SINA sequences, trimmed according to Methods. (FASTA 7 kb)

Additional file 12: Output from MEGA7 Model Selection to generate Fig. 1. Excel spreadsheet related to selection of phylogenetic models for Fig. 1 tree**,** sorted by AICc values. (XLS 525 kb)

Additional file 13: Sequence alignment of the four consensus sequences (SINA, SIAH1, SIAH2, SIAH3). Consists of 4 sequences generated from the refined subalignments (see Methods). (FASTA 1 kb)

## Abbreviations

DIMER: Dimerization
EGFR: Epidermal growth factor receptor
HER2: Human epidermal growth factor receptor 2
K-RAS: Kirsten rat sarcoma viral oncogene
LCA: Last common ancestor
MAPK: Mitogen-activated protein kinase
MEK: Mitogen-activated protein kinase kinase/extracellular signal-regulated kinase kinase (MAPK/ERK kinase).
RAF: Rapidly Accelerated Fibrosarcoma
RAS: Rat sarcoma viral oncogene
RING: Really Interesting New Gene
S3UNS: SIAH3-unique N-terminal sequences
SBD: Substrate binding domain
SBS: Substrate binding site
SEV: Sevenless
SIAH: SINA homolog
SINA: Seven-In-Absentia
SOS: Son of Sevenless
SZF: SIAH-type zinc finger
