# Supplementary material for: Phylogenetic analysis of the SINA/SIAH ubiquitin E3 ligase family in Metazoa
Source: BMC Evol Biol. 2017 Aug 7;17:182. doi: 10.1186/s12862-017-1024-x (PMC5547486; doi:10.1186/s12862-017-1024-x)
Supplement: Supplementary file 2 — Each SINA/SIAH sequence is linked with online accession by one click. List of SINA/SIAH protein sequences (n = 70) that were utilized in the phylogenetic analyses as presented in this study. Taxonomic designation for representative species (n = 39) is listed to the left of its name. With the exception of the sequence from Petromyzon marinus, which was acquired from UniProtKB, all amino acid sequence identifiers presented in this table refer to their NCBI Genbank accession numbers. Additional file 2: Table S1 is identical to Table 1, except that the online accession version of these SINA/SIAH sequences was included in the Additional file 2: Table S1. (DOCX 24 kb) [file 12862_2017_1024_MOESM2_ESM.docx]

|  | **Taxonomy** | **Species** | **SIAH1** | **SIAH2** | **SIAH3** |
| --- | --- | --- | --- | --- | --- |
|  |  | ***Vertebrates*** |  |  |  |
| 1 | Mammalia | Homo sapiens | NP_003022.3 | NP_005058.3 | NP_942146.2 |
| 2 | Mammalia | Pan troglodytes | NP_001233288.1 | XP_516819.2 | XP_522672.3 |
| 3 | Mammalia | Mus musculus | NP_033198.1 | NP_033200.2 | NP_001121565.1 |
| 4 | Mammalia | Bos taurus | XP_005218736.1 | NP_001193983.1 | NP_001192350.1 |
| 5 | Mammalia | Tursiops truncatus | XP_019788031.1 | XP_019789908.1 | XP_019792000.1 |
| 6 | Mammalia | Monodelphis domestica | XP_007474893.1 | XP_001363407.1 | XP_007501643.1 |
| 7 | Aves | Gallus gallus | XP_015147897.1 | XP_426719.2 | XP_417044.1 |
| 8 | Aves | Sturnus vulgaris | XP_014738058.1 | XP_014741604.1 | XP_014750862.1 |
| 9 | Reptilia | Pogona vitticeps | XP_020643987.1 | XP_020637543.1 |  |
| 10 | Reptilia | Thamnophis sirtalis | XP_013913965.1 | XP_013927960.1 |  |
| 11 | Reptilia | Alligator mississippiensis | XP_019352994.1 | XP_006259223.1 | XP_019356083.1 |
| 12 | Reptilia | Chrysemys picta | XP_008165194.1 | XP_005286979.1 | XP_005287220.1 |
| 13 | Amphibia | Xenopus tropicalis | NP_001015836.1 | NP_001095281.1 | XP_002941011.1 |
| 14 | Sacropterygii | Latimeria chalumnae | XP_005998413.1 | XP_006008925.1 | XP_014352065.1 |
| 15 | Neopterygii | Danio rerio | NP_955815.1 | NP_956721.2 |  |
| 16 | Neopterygii | Salmo salar | XP_013979721.1 | XP_014052924.1 |  |
| 17 | Neopterygii | Oreochromis niloticus | XP_019217515.1 | XP_003459581.3 |  |
| 18 | Chondrichthyes | Callorhinchus milii | XP_007887616.1 | XP_007889716.1 | XP_007889930.1 |
| 19 | Cyclostomata | Petromyzon marinus | S4R9G1_PETMA |  |  |
|  |  | ***Invertebrates*** | **SINA** |  |  |
| 20 | Cephalochordata | Branchiostoma floridae | XP_002609562.1 |  |  |
| 21 | Echinodermata | Strongylocentrotus purpuratus | XP_797311.2 |  |  |
| 22 | Arthropoda | Drosophila melanogaster | NP_476725.1 |  |  |
| 23 | Arthropoda | Anopheles gambiae | XP_001688791.1 |  |  |
| 24 | Arthropoda | Apis mellifera | XP_394284.2 |  |  |
| 25 | Arthropoda | Acyrthosiphon pisum | XP_008186354.1 |  |  |
| 26 | Nematoda | Caenorhabditis elegans | NP_500409.1 |  |  |
| 27 | Nematoda | Necator americanus | XP_013306181.1 |  |  |
| 28 | Nematoda | Brugia malayi | XP_001898781.1 |  |  |
| 29 | Nematoda | Trichinella spiralis | XP_003379392.1 |  |  |
| 30 | Spiralia | Octopus bimaculoides | XP_014779248.1 |  |  |
| 31 | Spiralia | Crassostrea gigas | XP_011434753.1 |  |  |
| 32 | Spiralia | Helobdella robusta | XP_009028819.1 |  |  |
| 33 | Spiralia | Schistosoma mansoni | XP_018646300.1 |  |  |
| 34 | Cnidaria | Nematostella vectensis | XP_001637064.1 |  |  |
| 35 | Cnidaria | Orbicella faveolata | XP_020623250.1 |  |  |
| 36 | Cnidaria | Acropora digitifera | XP_015766642.1 |  |  |
| 37 | Cnidaria | Hydra vulgaris | XP_002162099.1 |  |  |
| 38 | Porifera | Amphimedon queenslandica | XP_019850617.1 |  |  |
| 39 | Placozoa | Trichoplax adhaerens | XP_002108034.1 |  |  |

**Supplemental** T**able S1.** List of sequences (n = 70) that were utilized in all evolutionary analyses as conducted in this study. Taxonomic designation for representative species (n = 39) is listed to the left of its name. With the exception of the sequence from *Petromyzon marinus*, which was acquired from UniProtKB, all amino acid sequence identifiers presented in this table refer to their NCBI Genbank accession numbers. Supplemental Table 1 is identical to Table 1, except that the accession version of these sequences was included in the Supplemental Table S1.
